# Do Grading Gray Stimuli Help to Encode Letter Position?

**DOI:** 10.3390/vision5010012

**Published:** 2021-03-04

**Authors:** Manuel Perea, Ana Baciero, Ana Marcet, María Fernández-López, Pablo Gómez

**Affiliations:** 1 Departamento de Metodología and ERI-Lectura, Universitat de València, 46010 Valencia, Spain; maria.fernandez@uv.es; 2 Centro de Ciencia Cognitiva, Universidad Antonio de Nebrija, 28015 Madrid, Spain; abaciero@nebrija.es; 3 Departamento de Didáctica de la Lengua y la Literatura, Universitat de València, 46022 Valencia, Spain; ana.marcet@uv.es; 4 Department of Psychology, Palm Desert Campus, California State University, San Bernardino, CA 92407, USA; pablo.gomez@csusb.edu

**Keywords:** word recognition, letter position coding, perceptual factors, lexical decision, orthographic processing

## Abstract

Numerous experiments in the past decades recurrently showed that a transposed-letter pseudoword (e.g., JUGDE) is much more wordlike than a replacement-letter control (e.g., JUPTE). Critically, there is an ongoing debate as to whether this effect arises at a perceptual level (e.g., perceptual uncertainty at assigning letter position of an array of visual objects) or at an abstract language-specific level (e.g., via a level of “open bigrams” between the letter and word levels). Here, we designed an experiment to test the limits of perceptual accounts of letter position coding. The stimuli in a lexical decision task were presented either with a homogeneous letter intensity or with a graded gray intensity, which indicated an unambiguous letter order. The pseudowords were either transposed-letter pseudowords or replaced-letter pseudowords (e.g., jugde vs. jupte). The results showed much longer response times and substantially more errors in the transposed-letter pseudowords than in the replacement-letter pseudowords, regardless of visual format. These findings favor the idea that language-specific orthographic element factors play an essential role when encoding letter position during word recognition.

## 1. Introduction

An experimental phenomenon that revolutionized the front-end of models of visual word recognition is the transposed-letter effect. The transposed-letter effect is defined as the difference in performance (i.e., response times and errors rates) between the transposed-letter and replaced-letter pseudowords (e.g., JUGDE is more wordlike than its control JUPTE) [1,2,3]. The transposed-letter effect is used to rule out the fixed slot coding scheme of the influential interactive activation model [4] and is the driving force behind many visual word recognition models, with more flexible input coding schemes for the letter position. Some of these models (e.g., overlap model [5]) stress the perceptual uncertainty due to the limitations of the visual system, when coding the letter position (e.g., the letters G and D in JUGDE would also activate the nearby positions, thus creating a percept of the word JUDGE)—note that these models have their roots in more general models of visual attention [6]. In contrast, other models (e.g., open bigram models) focus on a language-specific, orthographic component, based on an “open bigram” level between the letter and word layers [7,8])—not that JUGDE would share all its open bigrams with JUDGE [JU, JD, JG, JE, UD, UG; UE, DE, GE], except for JD/DG.

Despite dozens of experiments on the transposed-letter effects (for review [9]), there is still no consensus on whether the perceptual or the orthographic accounts provide a better interpretation of the findings. Some findings appear to favor perceptual accounts—transposed-letter effects are minimal in a non-visual tactile modality like braille [10]—keeping in mind that orthographic processing with braille words and printed words is quite similar [11]. In contrast, other findings appear to favor the orthographic account—transposed-letter effects are greater for letter strings than for strings of different types of objects (e.g., numbers, symbols, [12,13]), presumably because of orthographic-specific processing.

One direct way to test the explanatory power of perceptual accounts of letter position coding is by manipulating visuoperceptual elements. This was the strategy followed by Marcet et al. [14] in a series of lexical decision experiments (i.e., deciding whether the presented item is a word or not). The idea was that highlighting the critical letters or presenting the letters serially would make them easily noticeable and thus reduce the perceptual uncertainty regarding the encoding of letter position. On a similar vein, previous research showed that the encoding letter identity (i.e., another component of orthographic processing) could be modulated by the visuoperceptual elements. For instance, in lexical decision, Grainger et al. [15] found that the neighborhood frequency effect (i.e., the slower identification for SPICE than for SAUCE due to the higher frequency neighbor SPACE) was greatly reduced when participants fixated on the disambiguating letter (i.e., I in SPICE).

Returning to the issue of the letter position coding, if the transposed-letter effect was drastically reduced with a visuoperceptual manipulation, this would favor perceptual explanations of letter position coding over orthographic accounts. Alternatively, if the transposed-letter effect is immune to a visuoperceptual manipulation, this would pose great difficulties on the perceptual explanations of letter position coding and it would definitely favor the orthographic accounts. To test these accounts, in Marcet et al.’s [14] second experiment, the items could be presented either with the critical transposed/replaced letters highlighted or not (e.g., CHOLOCATE; CHOLOCATE). Marcet et al. [14] found a reduction in the magnitude of the transposed-letter effect, relative to the standard format. The transposed-letter effect was still quite large for the highlighted pseudowords (109 ms in the latency data; more than 14.6% in the error data). In their third experiment, the letters were presented serially, one at a time—each letter was presented for 200 ms in this corresponding relative position. The idea was that, using this serial procedure, the letters in the string could not be processed in parallel, thus it would resemble braille reading. While the transposed-letter effect in the serial format was smaller than in the standard “immediate” format, the size of the effect in the serial presentation was quite large (75 ms in the latency data; more than 27.9% in the error data). Following Massol et al. [12], Marcet et al. [14] concluded that the transposed-letter effect had both a perceptual locus (common to all visual objects) and an orthographic locus (specific to letter strings).

**Figure 1:**
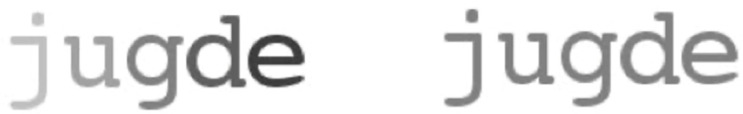
An instance of a graded gray format (**left**) and a homogeneous format (**right**) for the transposed-letter pseudoword jugde.

In the present paper, we employed a novel perceptual manipulation to test the limits of the perceptual accounts of letter transposition effects. The idea was to combine a visuoperceptual element (namely, visual intensity) with the letter order. Specifically, each letter of an item had progressively more intensity (i.e., grading gray manipulation; see left panel of Figure 1). For the homogeneous format, we chose a constant intermediate intensity of the letters (see top panel of Figure 1).

The idea of the manipulation was that the grayscale might work as feature that facilitated letter position coding; in the example from Figure 1, the **g** is not only to the left of **d** but is also lighter. Clearly, if participants encode the letter ordering information coming from the increasing intensity of the letters while reading words, one would expect a smaller transposed-letter effect than in the control, homogeneous condition. The rationale was that, when processing a letter string, this grading gray manipulation might help better establish the locational gradient that underlay the letter position coding (see Figure 2 in [8] for an example). Thus, at an empirical level, we explored the robustness of the transposed-letter effect to a visuospatial manipulation; and at a theoretical level, the present experiment tested whether the transposed-letter effect diminished relatively to a homogeneous gray format, as a perceptual account would predict. Alternatively, an orthographic account would posit that the transposed-letter effect would be independent of the format.

Besides its theoretical implications for visual word recognition models, our visuoperceptual manipulation of the letter order might (if successful) be useful to help with reading in individuals with letter position dyslexia. This is a deficit that affects how readers encode letter position within words [16]. If the gray grading manipulation diminishes the transposed-letter effect, one might use this manipulation in text reading, for individuals with letter position dyslexia. Thus, the gray grading manipulation could complement, at the level of letter position coding, the dyslexia-friendly typefaces that are currently available for the level of letter identity coding [17].

## 2. Materials and Methods

### 2.1. Participants

The participants were 36 DePaul University undergraduate students (mean age = 20.2 years old; range: 18–26), all native speakers of English. They had normal (corrected) vision and signed a consent form before the experiment. None of the participants reported having reading problems.

### 2.2. Materials

We employed the set of 120 transposed-letter pseudowords (e.g., jugde; baseword: judge) and replacement-letter pseudowords (e.g., jupte) from [1]. All stimuli were 5 letters in length. The mean baseword frequency was 15.6 per million words (range: 0.1–97.3) and the mean orthographic Levenshtein distance OLD20 (i.e., a measure of orthographic density) was 1.7 (range: 1.3–2.4) in Balota et al.’s [18] English Lexicon Project. A set of 120 words of similar length as that of the pseudowords was used for the purposes of lexical decision (mean word frequency = 91.2 per million [range: 9.1–269.4]; mean OLD20 = 1.7 [range: 1.1–2.4]). The entire list of items (words, transposed-letter pseudowords, replacement-letter pseudowords) is presented in Appendix A. To create the images for the grading gray and the homogeneous stimuli, we wrote a routine in R—the code was presented in Appendix B. We created four experimental lists composed of 240 trials, to counterbalance the stimuli across all four conditions, following a Latin Square design. For instance, List 1 would present the transposed-letter pseudoword jugde in grading gray; List 2 would present the transposed-letter pseudoword jugde in homogenous format in List 2; List 3 would present the replacement-letter pseudoword jupte in grading gray; and List 4 would present the replacement-letter pseudoword jupte in a homogeneous format. Each list was composed of 120 words, 60 transposed-letter pseudowords (30 in grading gray format; 30 in homogeneous format), and 60 replacement-letter pseudowords (30 in grading gray format; 30 in homogeneous format).

### 2.3. Procedure

Testing took place in a quiet room. We employed DMDX [19] with Windows-based computers to present the stimuli and collect the responses. On each trial, a 500-ms fixation point (“+”) preceded the stimulus item’s presentation. The item was on the screen until response—or when a 2000-ms deadline passed. Participants had to decide whether the stimulus was a word in English or not, by pressing the “yes” key or the “no” key. The instructions stressed both speed and accuracy (i.e., “be as fast as possible but trying to keep low the number of errors”). A practice list composed of 16 trials (8 words, 4 transposed-letter pseudowords, 4 replacement-letter pseudowords) preceded by 240 experimental trials. There were short breaks every 80 trials. The session lasted approximately 15 min.

## 3. Results

**Figure 2:**
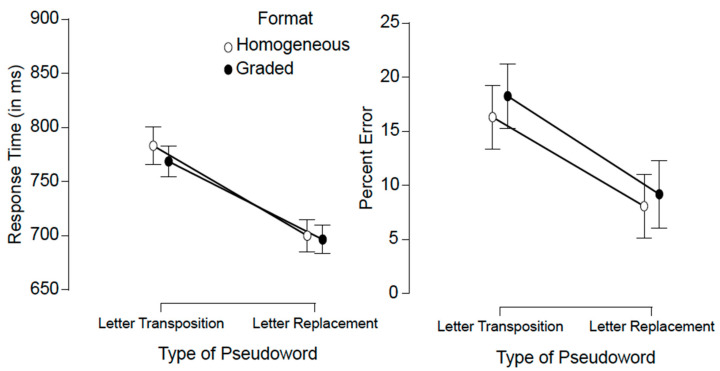
Mean RTs and error rates in each of the conditions of the experiment for the pseudowords. The bars represent the standard errors.

For the analyses of the response time (RT) data, we excluded the error responses (12.9% for pseudowords; 7.4% for words) and the latencies that were shorter than 250 ms (less than 0.1%). As the deadline for a response was set to 2000 ms, correct RTs could not be longer than the said deadline. Figure 2 presents the averages per condition (with the bars representing the standard errors) for each of the pseudoword stimuli conditions.

To analyze the pseudoword data, we conducted frequentist and Bayesian ANOVAs in JASP 0.14.1 [20] on the participants’ means per condition with Type of Pseudoword (Letter Transposition, Letter Replacement) and Format (Graded, Homogeneous) as fixed Factors. Importantly, the Bayesian ANOVAs allowed us to measure the likelihood of the null vs. alternative hypotheses, given the data. For instance, a BF_10_ value of 5 would be interpreted as the alternative hypothesis being 10 times more likely than the null hypothesis, with these dataset (e.g., BF_10_ > 3 would be interpreted as “substantial evidence” in favor of the alternative hypothesis—note that BF_10_ values less than 1 reflect evidence toward the null hypothesis. To analyze the word data, the only fixed factor was Format (Graded, Homogeneous). Of note, we present ANOVAs rather than linear mixed-effects models for simplicity—needless to say, the analyses using these models produced the same pattern of findings.

### 3.1. Word Data

Lexical decision times were longer for the graded gray words than for the homogeneous words (657 vs. 639 ms), F(1.36) = 8,27, MSE = 689.3, *p* = 0.007, BF_10_ = 6.74. Error rates were only slightly higher for the graded gray than for the homogeneous words (7.7 vs. 7.0%), F(1.36) = 2.31, MSE = 3.28, *p* = 0.14, BF_10_ = 0.62.

### 3.2. Nonword Data

Lexical decision times were substantially longer for the transposed-letter pseudowords than for the replacement-letter pseudowords (see Figure 2), F(1.36) = 102.54, MSE = 2120.7, *p* < 0.001, BF_10_ = 8.67 × 10^11^. Neither the main effect of format nor the interaction between the two factors approached significance (both Fs < 1; format: BF_10_ = 0.296; interaction: BF_10_ = 0.282).

We found a greater percentage of errors for the transposed-letter pseudowords than for the replacement-letter pseudowords (see Figure 2), F(1.36) = 35.35, MSE = 76.32, *p* < 0.001, BF_10_ = 2.21 × 10^9^. The effect of format barely reached the significance level, F(1.36) = 4.51, MSE = 18.62, *p* = 0.041, BF_10_ = 0.44, and, more importantly, there were no signs of an interaction between the two factors, F < 1, BF_10_ = 0.24.

## 4. Discussion

The main aim of the present experiment was to test whether a visuoperceptual manipulation that was perfectly correlated to letter order (a graded gray manipulation) could help encode letter position—serving as an additional letter order cue—as compared to a homogeneous gray condition (see Figure 1). The rationale behind this manipulation was that the letter position and letter identity could be thought as perceptual features to be bound, and hence binding errors are possible. By adding an extra perceptual cue—the gray tone, we hypothesized that the transposed letter effect could be attenuated. The results do not show such attenuation. First, the visuoperceptual manipulation had a harmful impact on the word stimuli (i.e., faster responding in the homogeneous than in the graded gray format). Second, and more importantly, we found no signs of a modulation of the transposed-letter effect for pseudowords as a function of visual format. Indeed, the Bayes Factors revealed that the data were most consistent when there was a lack of interaction between the two variables.

The present experiment represents yet another demonstration of the difficulty of drastically reducing the transposed-letter effect using a visuoperceptual manipulation. As stated in the Introduction, Marcet et al. [14] found a slight decrement in the transposed-letter effect when the critical letters were highlighted (e.g., CHOLOCATE) relative to the standard format—note, however, that the transposed-letter effect for the highlighted pseudowords was still quite large. Here, by using a visuoperceptual manipulation that offered precise information of letter order (i.e., the initial letters were in a less pronounced intensity than the final letters; see Figure 1), we found no signs of better encoding of letter position for the pseudoword stimuli—further, this was accompanied by some processing cost for the word stimuli.

Our findings have three clear-cut implications. At a theoretical level, they favor the view that a critical component of letter position coding occurs at an abstract level [12,14]. Thus, we believe that letter position encoding might be better conceptualized as implying several processing stages—from an early more perceptual process (presumably via perceptual noise shared with other visual objects) to a later orthographically-specific process. There are currently hybrid models that take elements from both perceptual and orthographic accounts [21]. At a methodological level, a potential avenue of the grading gray manipulation would be to test how it might interact with the serial position function of letter positions in target-in-string tasks, where the letter string is presented for a limited time (less than 100 ms) and participants have to indicate the identity of one of the letters—note that for a homogenous format, previous research has typically showed a W-shaped function in accuracy (see [13,22]). Finally, at a more practical level, the similar performance for the transposed-letter pseudowords like JUGDE and JUDGE in skilled adult readers suggests that the grading gray perceptual manipulation might not help reading in individuals with letter position dyslexia. Indeed, the word data indicate that our visuoperceptual manipulation might impair word processing with no apparent benefit.

## Data Availability

The dataset and scripts are available at the OSF link: https://osf.io/x2y5g/?view_only=a5948acf67fd40d4aa8c8aeda560b386 (accessed on 25 December 25 2020).

## Appendix A

List of stimuli in the experiment

Words: boost; solve; kings; slave; porch; risen; stock; brave; loath; rally; steer; brief; likes; habit; drums; sides; pride; exile; marks; sword; swept; third; lyric; brass; ponds; paint; graph; sixty; glass; smash; brick; tails; bound; slots; medal; alien; sorry; faith; glare; angle; crook; cover; crisp; ankle; arrow; purse; means; haste; debts; kneel; barge; flash; bills; heard; taxes; agree; hosts; prone; merit; views; fence; shape; signs; inner; basic; squad; think; blink; bears; spent; shade; flags; field; slope; rules; great; light; apart; craft; lunch; hairy; count; crawl; store; flock; terms; slice; reply; agent; drama; woman; pound; ivory; spoon; sharp; begin; asked; raise; scare; thing; grant; front; crowd; scene; parks; union; smart; state; enter; apple; shame; ranks; stink; guess; limit; water; track; loose; young; stage.

Pseudowords (TL-pseudowords, RL-pseudowords): loewr, loanr; golve, gatve; spary, sposy; tlels, tfols; selet, satet; celrk, cohrk; mienr, miosr; scraf, scnef; laeks, laots; cuoch, caech; rievr, rianr; sener, sacer; colck, catck; maigc, maoyc; cluod, clead; trian, troen; jials, jeols; friut, freot; cions, cuens; ruond, reand; nedes, netas; hevay, henoy; pliot, pfeot; gievn, giosn; paerl, puorl; banrs, bascs; baord, buerd; sutdy, sehdy; sitck, sofck; faecs, faons; muont, maent; hlils, hfels; macrh, masnh; fanit, faset; faatl, faefl; cerek, cusek; forwn, fenwn; anrgy, anspy; toewr, tounr; abvoe, abnue; mokns, motcs; ruogh, reagh; bahte, bakfe; piont, peant; whloe, whtae; litfs, likls; tihef, tefef; sheos, shuas; botls, bokfs; haeds, houds; skrit, sknot; culry, cufny; palnt, pofnt; awkae, awfoe; bilbe, bitde; shvae, shnoe; sehep, sofep; tihgt, titpt; ocaen, ocoun; muisc, muonc; idaes, idous; soebr, soafr; shrot, shnet; snogs, scegs; jugde, jupte; waogn, waujn; meatl, meokl; vaule, vaote; gilrs, gifcs; setms, sohms; palys, pokys; raost, reust; paech, puoch; sohot, sekot; chlid, chfod; roibn, roodn; taech, toich; maojr, maegr; tiegr, tiopr; loevs, loons; borom, bunom; coarl, counl; lagre, lapne; emtpy, emljy; ealry, eatny; mabye, mafge; notrh, nodnh; oinon, oecon; kinfe, kosfe; chiar, choer; leomn, leasn; roayl, roepl; spaek, spuok; maets, mouts; braed, bruod; sipce, sagce; patns, palcs; fiary, foery; riigd, riopd; selep, sofep; uhser, ufner; paepr, paogr; huose, hease; balck, botck; neevr, neanr; teteh, telah; claen, cloun; flaes, fluos; caeml, caorl; fruad, froed; baocn, bausn; golbe, gutbe; slels, sfols; crary, cnory; pokla, pofta; nusre, nunce; felsh, fofsh; croks, cneks; unlce, untce; satly, safky.

## Appendix B

Fuction to create the grading gray and homogenous stimuli.
